# Prediction of onset of remnant gastric cancer by promoter DNA methylation of *CDO1*/*HOPX*/*Reprimo*/*E-cadherin*

**DOI:** 10.18632/oncotarget.26814

**Published:** 2019-03-29

**Authors:** Keita Kojima, Naoko Minatani, Hideki Ushiku, Satoru Ishii, Toshimichi Tanaka, Keigo Yokoi, Nobuyuki Nishizawa, Yosuke Ooizumi, Kazuharu Igarashi, Kei Hosoda, Hiromitsu Moriya, Hiroaki Mieno, Masahiko Watanabe, Keishi Yamashita

**Affiliations:** ^1^ Department of Surgery, Kitasato University School of Medicine, Sagamihara, Kanagawa 252-0329, Japan; ^2^ Division of Advanced Surgical Oncology, Research and Development Center for New Frontier, Kitasato University School of Medicine, Sagamihara, Kanagawa 252-0329, Japan

**Keywords:** remnant gastric cancer, cysteine dioxygenase 1 (CDO1), homeodomain-only protein X (HOPX), Reprimo, E-cadherin

## Abstract

**Background:**

Early detection of remnant gastric cancer (RGC) is required to reduce the risk of death, but long-term endoscopic surveillance is difficult after gastrectomy. In this study, data for the methylation status of 4 methylation genes (*CDO1, HOPX, Reprimo,* and *E-cadherin*) to predict the onset of RGC are presented.

**Results:**

The 4 genes showed hypermethylation in RGC tumors in contrast to the corresponding non-cancerous mucosa tissues. The methylation level in the non-cancerous mucosa tissues of the initial surgery was obviously high in initial malignant disease for *CDO1* (*P* = 0.0001), while in initial benign one for *E-cadherin* (*P* = 0.003). Promoter DNA methylation status in the remnant non-cancerous mucosa tissues together with the basic clinical data in turn predicted either initial malignant disease or initial benign disease with a high AUC score of 0.94, suggesting that methylation events are differentially recognized between the initial malignant and benign disease. We then finally confirmed that 4 genes hypermethylation of the non-cancerous tissues by biopsy prior to onset of RGC could predict terms until RGC occurred (*P* < 0.0001).

**Methods:**

A total of 58 RGC patients were used to establish the model. The 4 genes promoter methylation were analyzed for DNA obtained from the patient’s specimens using quantitative methylation specific polymerase chain reaction.

**Conclusions:**

This risk model would help provide guidance for endoscopic surveillance plan of RGC after gastrectomy.

## INTRODUCTION

Remnant gastric cancer (RGC) arises in the remnant stomach after gastrectomy [1]. The development of RGC has been considered to be influenced by extrinsic factors such as decreased motor function and bile reflux [2–5]. Billroth II reconstruction has been reported to carry an increased risk of RGC because of bile reflux [6]. Other intrinsic factors including *Helicobacter pylori* (HP) infection [7], *Epstein-Barr* virus infection [8], and microsatellite instability [9] are also implicated in the development of RGC.

RGC is usually detected at an advanced stage and is thus associated with a low rate of radical resection and poor outcomes [7], and its early detection is essential to improve prognosis. The prevalence of RGC has been reported to be 2.1% [10]. Periodic endoscopic examinations are recommended every 2 to 5 years for at least 20 years after gastrectomy [11], while almost patients have discouraged such periodic endoscopic examinations due to poor cost-effectiveness [3, 12]. Continued surveillance imposes considerable stress on patients and increases healthcare costs. Markers to predict the risk of RGC are highly demanded.

DNA methylation often correlates with the risk of carcinogenesis [13, 14]. Epigenetic abnormalities may have already accumulated in apparently normal tissue, leading to “epigenetic field cancerization” associated with an increased risk of carcinogenesis [15, 16]. In the present study, epigenetic field cancerization was assessed in non-cancerous sites for cysteine dioxygenase 1 (*CDO1*), homeodomain-only protein X (*HOPX*), *Reprimo*, and *E-cadherin*, because they were cancer-specific and frequent aberrations in gastric cancer [17–21].

The *CDO1* is an enzyme that converts cysteine to cysteine sulfinic acid to augment reactive oxygen species generation [17]. *HOPX* was discovered as an essential gene for development of the heart [22], and also functions as a tumor suppressor gene through Wnt inhibition [23–25]. The *Reprimo* regulates the cell cycle [26–28]. The *E-cadherin* plays an important role in cell adhesion among epithelial cells, and its mutations cause hereditary diffuse gastric cancer [29] and sporadic gastric cancer [30].

In this paper, we report that methylation of these 4 genes can predict short-term development of RGC on the basis of biopsy specimens obtained by endoscopy.

## RESULTS

### Clinicopathological characteristics of patients with RGC

Among patients who underwent gastrectomy for RGC, 23 had benign disease, and 35 had malignant disease at the initial diagnosis. The most outstanding clinicopathological characteristic was the term from the initial operation to onset of RGC, which was significantly shorter in patients with malignant disease (191.5 ± 127.5 months) than in those with benign disease (485.7 ± 86.7 months; *P* < 0.0001). HP infection status was available in only 11 patients. The other clinicopathological characteristics of the patients are shown in Supplementary Table 1.

### Analysis of the methylation level of the promoter DNA of the 4 methylation genes in RGC tissues and the corresponding non-cancerous mucosa tissues

**Figure 1:**
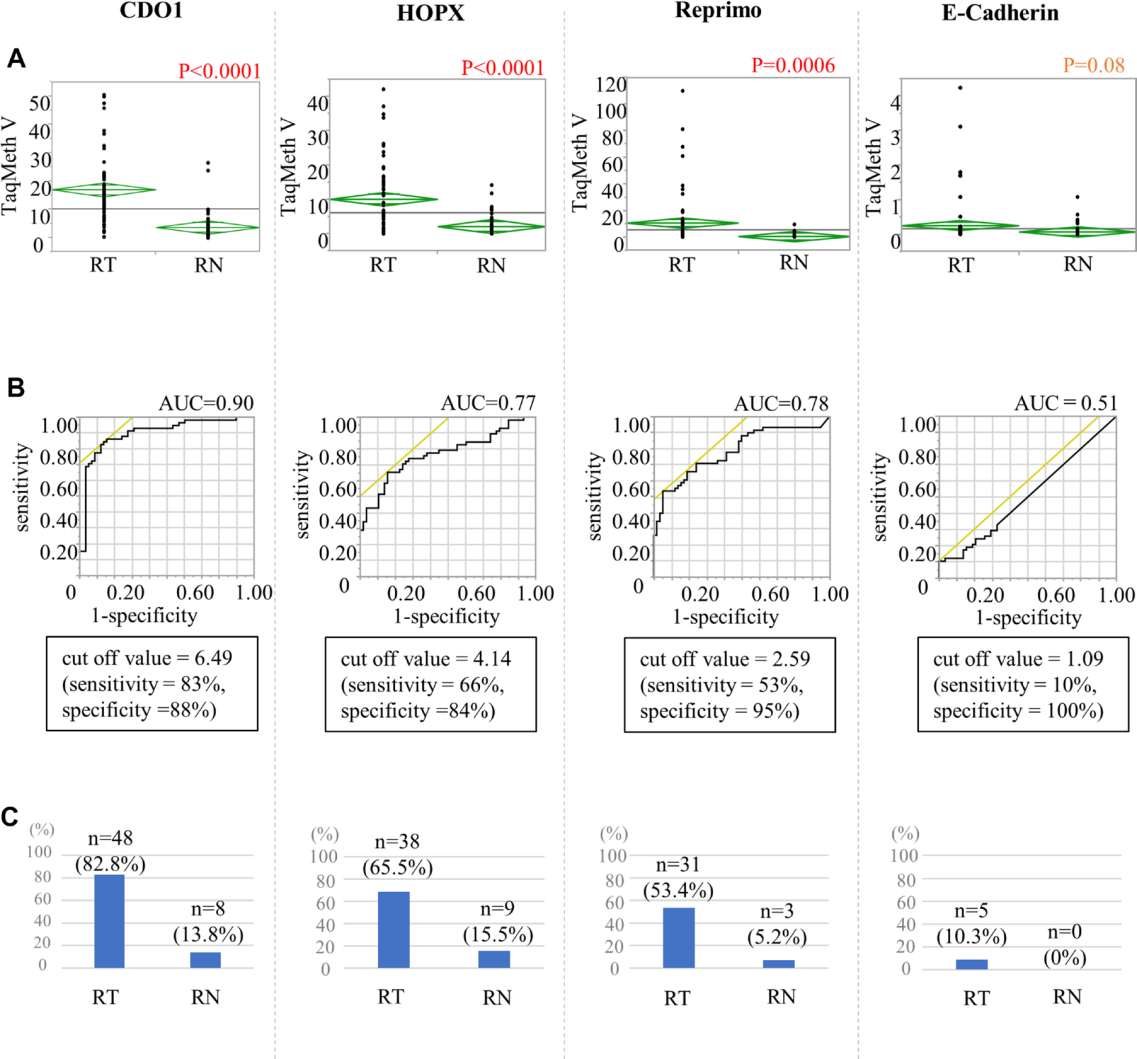
The calculation of the cut-off value of the cancer tissue and non-cancerous mucosa in the remnant stomach (**A**) Comparison between remnant gastric cancer tissue (RT) and remnant stomach non-cancerous mucosa (RN) using a *t*-test. (**B**) ROC curve for distinguishing between RT and RN. (**C**) The proportions of patients with TaqMeth Vs higher than the cut-off values.

First, the degree of hypermethylation of the specific DNA promoter regions of the 4 methylation genes (*CDO1*, *HOPX*, *Reprimo*, and *E-cadherin*) was assessed in tumor tissues and the corresponding non-cancerous mucosa tissues in 58 RGC patients (Figure 1A). TaqMeth Vs were significantly higher in the tumor tissues than in the corresponding non-cancerous mucosa tissues for *CDO1* (*P* < 0.0001), *HOPX* (*P* < 0.0001), and *Reprimo* (*P* = 0.0006), except for the *E-cadherin* (*P* = 0.08).

Optimal cut-off values of TaqMeth Vs for the 4 genes to distinguish the tumor tissues from the corresponding non-cancerous mucosa tissues were determined in RGC; 6.49 for *CDO1*, 4.14 for *HOPX*, 2.59 for *Reprimo*, and 1.09 for *E-cadherin*. As a result, the proportions of patients with TaqMeth Vs higher than the cut-off values were 82.8% and 13.8% for *CDO1*, 65.5% and 15.5% for *HOPX*, 53.4% and 5.2% for *Reprimo*, and 10.3% and 0% for *E-cadherin*, respectively (Figure 1B, 1C).

Next, clinicopathological characteristics were examined according to TaqMeth Vs for the 4 genes in RGC tissues and the corresponding non-cancerous mucosa tissues. The mean TaqMeth V in RGC tissues was 16.90 ± 12.08 for *CDO1*, 10.07 ± 10.56 for *HOPX*, 10.63 ± 21.43 for *Reprimo*, and 0.26 ± 0.77 for *E-cadherin*. The relationships of the TaqMeth Vs of the 4 genes in RGC tissues to the clinicopathological characteristics are shown in Table 1 and Supplementary Figure 1A and 1B. The TaqMeth V for *CDO1* gene differed significantly between patients with (y)pStage ≤II and those with (y)pStage ≥III (*P* = 0.04). *E-cadherin* differed significantly according to the presence or absence of lymph node metastasis (*P* = 0.01).

**Table 1:** Relationship with TaqMeth V of *CDO1*, *HOPX*, *Reprimo* and *E-cadherin* and clinicopathological factors at cancer tissue and non-cancerous mucosa

Clinicopathological factors	*n*	*P*-value^a^			
		*CDO1*	*HOPX*	*Reprimo*	*E-cadherin*
Age (≤70 : 70<)	29 : 29	0.39/0.34	0.31/0.14	0.09/0.98	0.66/0.99
Sex (M : F)	51 : 7	0.07/0.36	0.39/0.98	0.14/0.63	0.64/0.42
Initial diagnosis (Malignancy : Benign)	35 : 23	0.67/0.14	0.25/0.10	0.29/0.68	0.83/0.23
Helicobacter pylori (+ : –)	5 : 6	0.18/0.10	0.06/0.12	0.68/0.12	0.20/0.54
Reconstruction of initial operation (BI : BII : Other)	38 : 16 : 4	0.71/0.35	0.93/0.79	0.95/0.84	0.56/0.46
Site of recurrence (Anastomotic site : Non-anastomotic site)	30 : 28	0.14/0.65	0.87/0.14	0.20/0.31	0.12/0.15
Depth of tumor invasion (∼sm : mp∼)	29 : 29	0.85/0.21	0.56/0.51	0.17/0.58	0.75/0.59
Lymph node metastasis (+ : –)	45 : 13	0.35/0.30	0.36/0.23	0.26/0.38	0.01/0.64
Distant metastasis (+ : –)	2 : 56	0.32/0.70	0.13/0.41	0.55/0.61	0.63/0.62
(y)pStage (∼II : III∼)	46 : 12	0.04/0.33	0.37/0.35	0.06/0.37	0.21/0.71
Lymphatic invasion (+ : –)	23 : 35	0.43/0.20	0.37/0.20	0.20/0.37	0.13/0.51
Venous invasion (+ : –)	21 : 37	0.29/0.09	0.80/0.46	0.34/0.43	0.65/0.39
Lauren's histology (intestinal type : diffuse type)	29 : 29	0.36/0.34	0.29/0.01	0.39/0.51	0.19/0.22
Infiltrative pattern (a : b : c)	8 : 17 : 33	0.23/0.50	0.57/0.19	0.40/0.97	0.61/0.18

a: cancer tissue/non-cancerous mucosa

TaqMeth V was then explored clinicopathologically in the corresponding gastric non-cancerous mucosa tissues. The mean TaqMeth V was 3.71 ± 4.74 for *CDO1*, 2.13 ± 2.92 for *HOPX*, 0.62 ± 1.52 for *Reprimo*, and 0.081 ± 0.19 for *E-cadherin*. The results are shown in Table 1 and Supplementary Figure 1C–1E. *HOPX* TaqMeth V increased with increased age when age was considered a continuous variable (*P* = 0.02), and differed significantly according to histologic type (*P* = 0.008).

### Prognostic analysis in patients with RGC

To examine whether the TaqMeth Vs of the 4 genes in RGC tissue can predict prognosis, a log-rank plot analysis was performed to calculate the optimal cut-off values for overall survival (OS) and relapse-free survival (RFS). For *CDO1*, the optimal cut-off TaqMeth V was 26.7 for OS (*P* = 0.0002; relative risk, 3.67) and for RFS (*P* = 0.01; relative risk, 2.54). For *HOPX*, the optimal cut-off value was 23.7 for OS (*P* = 0.03; relative risk, 2.17), and no optimal cut-off value was obtained for RFS. For *Reprimo* and *E-cadherin*, no optimal cut-off value was obtained for either OS or RFS.

A univariate prognostic analysis of the clinicopathological factors and the obtained cut-off values for OS was performed using a log-rank test. The results showed that the T, N, M, (y)pStage, ly, v, INF, *CDO1* TaqMeth V, and *HOPX* TaqMeth V were significant prognostic factors for OS. Each TNM factor was excluded in the subsequent multivariate prognostic analysis. A multivariate Cox proportional hazards model was developed with the significant univariate prognostic factors. It was found that (y)pStage was the only independent prognostic factor, and *CDO1* TaqMeth V and *HOPX* TaqMeth V were eliminated as independent prognostic factors (Supplementary Table 2, Supplementary Figure 2A).

A univariate prognostic analysis was similarly performed for RFS and showed initial diseases (malignant or benign), T, N, M, (y)pStage, ly, v, INF, and *CDO1* TaqMeth V were significant prognostic factors for RFS. Multivariate prognostic analysis showed that (y)pStage and v were independent prognostic factors, *CDO1* TaqMeth V was eliminated (Supplementary Table 3, Supplementary Figure 2B).

### TaqMeth Vs of the 4 methylation genes in non-cancerous mucosa tissues at the initial surgery

Next, the methylation levels of the non-cancerous mucosa tissues at the initial surgery of the RGC patients were evaluated. In 17 patients with malignant disease at the initial diagnosis, the normal-appearing mucosal specimens obtained at the initial surgery were similar to those in patients with RGC. The TaqMeth Vs of the 4 genes were as follows: *CDO1*, 13.1 ± 13.3; *HOPX*, 3.6 ± 4.7; *Reprimo*, 0.8 ± 2.2; and *E-cadherin*, 0.1 ± 0.1.

In patients with an initial diagnosis of benign disease, non-cancerous mucosa tissues could not obtain. Therefore, tissue samples of non-cancerous mucosa obtained from 12 patients who underwent gastrectomy for gastric or duodenal ulcer in our hospital were substituted, and the methylation levels of the 4 genes were measured. The TaqMeth Vs were as follows: *CDO1*, 4.2 ± 5.6; *HOPX*, 1.2 ± 1.7; *Reprimo*, 0.5 ± 0.9; and *E-cadherin*, 0.3 ± 0.2.

**Figure 2:**
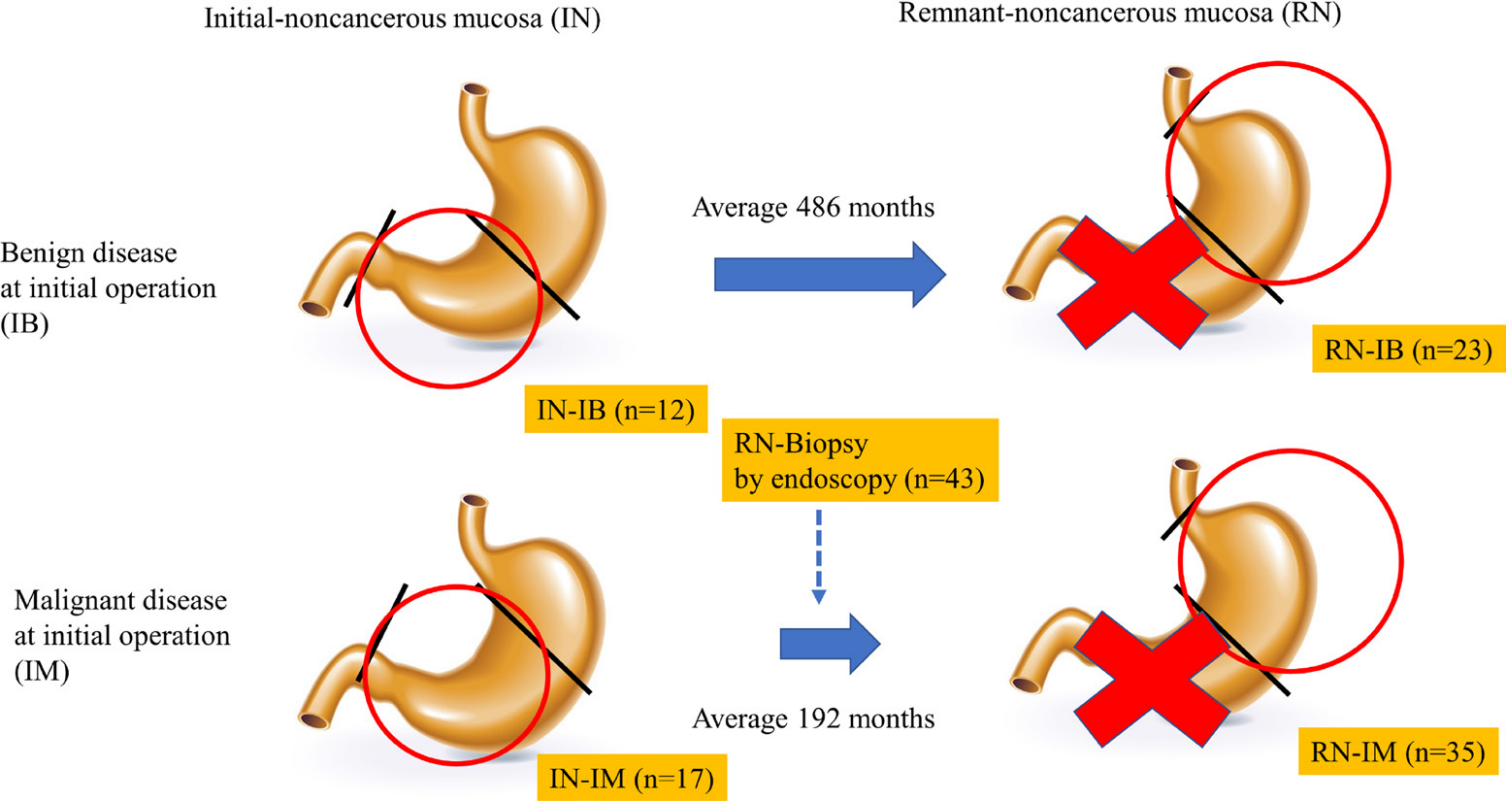
Scheme of the period of sampling non-cancerous mucosa Circle indicates the region where gastric mucosa was collected. We distinguished non-cancerous mucosa by diagnosis at initial surgery. We collected non-cancerous mucosa at the time of initial surgery and at the onset of remnant gastric cancer, respectively. We underwent endoscopic biopsy during follow-up from initial surgery.

**Figure 3:**
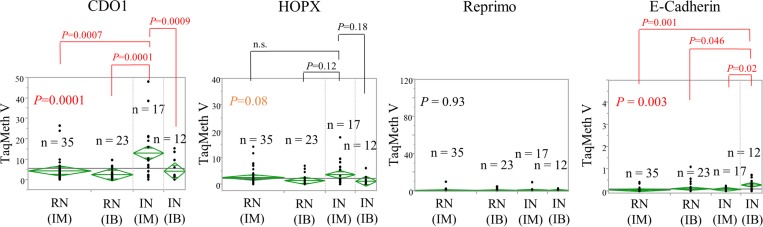
Variance analysis of TaqMeth V of each gene derived from remnant stomach or initial gastrectomy non-cancerous mucosa specimens whose first diagnoses were malignant or benign RN: remnant gastric non-cancerous mucosa. IN: initially obtained specimens of non-cancerous mucosa. IM: initial diagnosis of malignant disease. IB: initial diagnosis of benign disease.

To delineate the characteristics of methylation of each gene in the gastric mucosa, ANOVA was performed for the TaqMeth V of each gene in remnant gastric non-cancerous mucosa from patients with an initial diagnosis of malignant disease (RN-IM)(*n* = 35), remnant gastric non-cancerous mucosa from patients with an initial diagnosis of benign disease (RN-IB)(*n* = 23), initially obtained specimens of non-cancerous mucosa from patients with an initial diagnosis of malignant disease (IN-IM) (*n* = 17), and initially obtained specimens of non-cancerous mucosa from patients with an initial diagnosis of benign disease (IN-IB) (*n* = 12) (Figure 2). Significant differences in methylation levels were obtained for *CDO1* (*P* = 0.0001) and *E-cadherin* (*P* = 0.003) (Figure 3). The *CDO1* TaqMeth V was significantly higher in IN-IM than in all other specimens of non-cancerous mucosa (RN-IM, RN-IB, and IN-IB). The *E-cadherin* TaqMeth V was significantly higher in IN-IB than in all other specimens of non-cancerous mucosa (RN-IM, RB-IB, and IN-IM). No significant differences of methylation levels were obtained for *HOPX* (*P* = 0.08) and *Reprimo* (*P* = 0.93) among the 4 non-cancerous mucosa types.

### Mutual associations of the 4 methylation genes in the tissues

Associations of 2 genes from each of the 4 genes were separately evaluated in RGC tissues (Supplementary Figure 3A–3D), RN tissues (Supplementary Figure 3E–3H), IN-IB (Supplementary Figure 3I–3L), IN-IM (Supplementary Figure 3M–3P), and biopsy specimens (RN-Biopsy in Figure 3) (Supplementary Figure 3Q–3T). Close correlations were obtained for methylation levels between *CDO1* and *HOPX*, between *CDO1* and *Reprimo*, and between *HOPX* and *Reprimo* in each type of tissue specimen. On the other hand, the *E-cadherin* methylation did not correlate with any other genes in almost comparison.

### Evaluation of the initial diagnosis and clinicopathological characteristics

**Figure 4:**
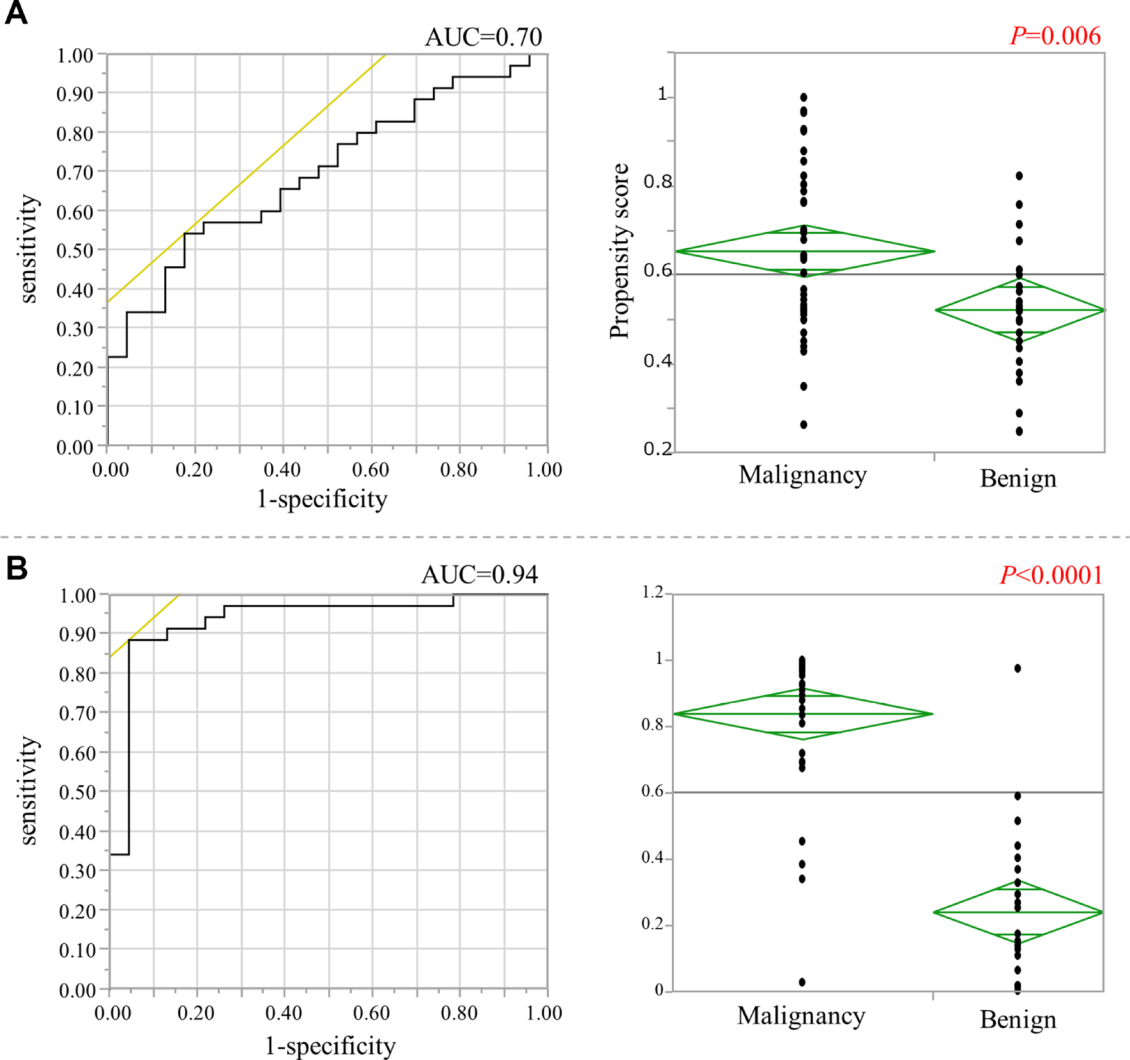
The calculation of the propensity scores for non-cancerous mucosa (**A**) (Left figure) ROC curve of the propensity score whose covariates were the TaqMeth Vs of the four genes derived from non-cancerous mucosa in remnant stomach. (Right figure) Comparison of PS between the two groups due to the difference in the initial diagnosis. (**B**) (Left figure) ROC curve of the propensity score whose covariates were the TaqMeth Vs of the four genes derived from non-cancerous mucosa in remnant stomach, age, sex, and time from the initial surgery. (Right figure) Comparison of propensity scores between the two groups due to the difference in the initial diagnosis.

Because the term from the initial operation to the RGC onset differed considerably according to the initial diagnosis of malignant disease and benign disease, the TaqMeth Vs of the 4 genes in remnant gastric non-cancerous mucosa (RN) were used as covariates and propensity scores were calculated with a logistic regression model (Figure 4A). The area under the receiver operating characteristic (ROC) curve was 0.7. The propensity scores were found to differ significantly according to whether the initial diagnosis was malignant disease or benign disease (*P* = 0.006).

Age, sex, and the time from the initial surgery to the RGC onset were then added as covariates, and the propensity scores were re-calculated (Figure 4B). The area under the ROC curve was 0.94. The propensity scores differed significantly between the groups (*P* < 0.0001), suggesting that onset of RGC could be reflected by the methylation levels of the RN in combination with patient basic data.

### Identification of predictors of the term to development of RGC

**Figure 5:**
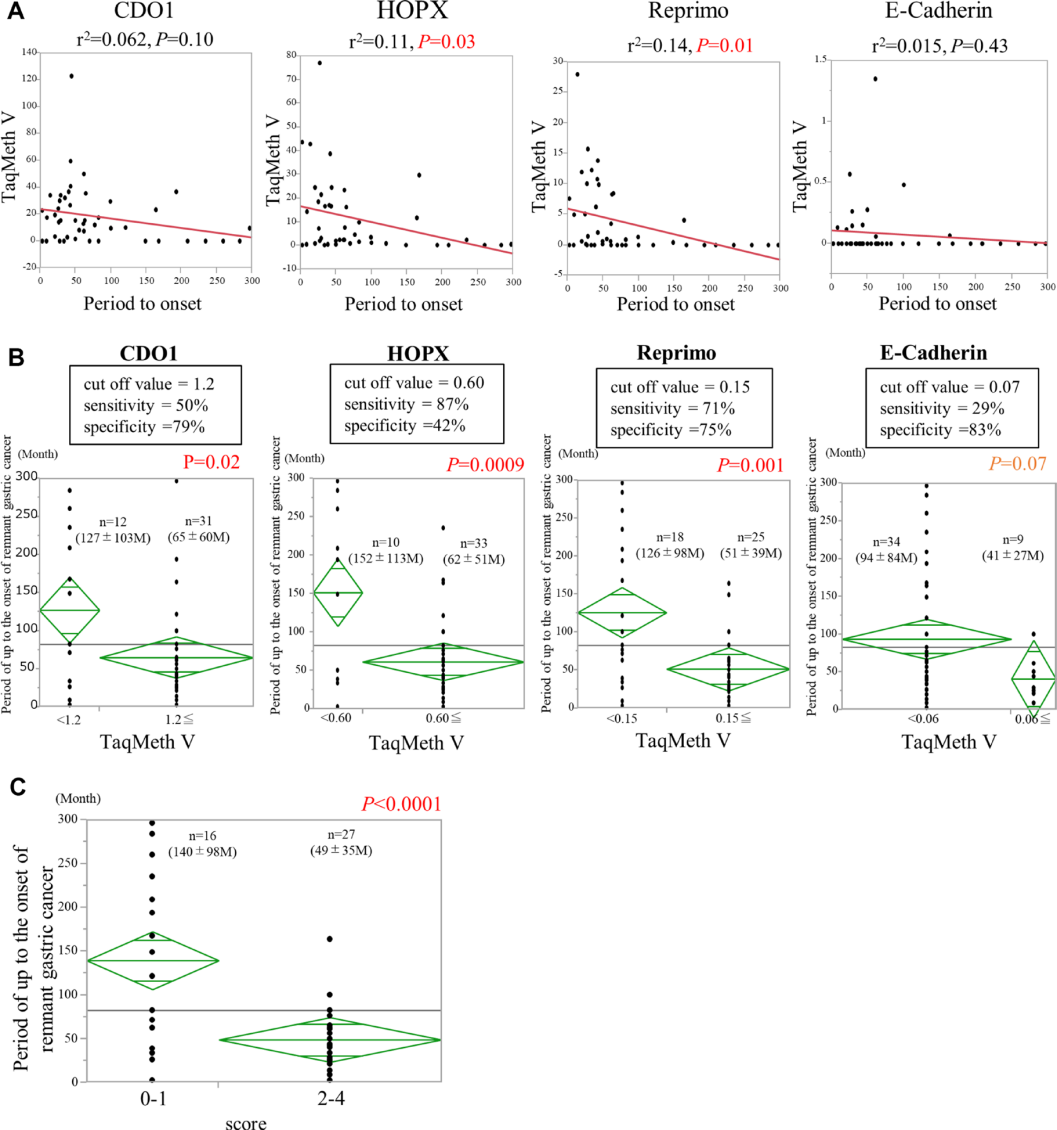
Predictors of the time to development of remnant gastric cancer (**A**) The relationships between the period to onset and the TaqMeth Vs of 4 genes. (**B**) Optimum cut-off TaqMeth V, sensitivity and specificity of each gene for dividing the time to development into a short-term group and a long-term group. Comparison with period to the onset of remnant gastric cancer between the two groups, which were divided by the cut-off value. (**C**) Comparison of the one point or less group with the two or more points group for the period to remaining gastric cancer onset.

To examine whether the short-term risk of the development of RGC could be predicted, the relationships of the TaqMeth Vs of the 4 genes in RN-Biopsy by Endoscopy obtained between the initial operation and the RGC onset were analyzed (Figure 5A). *CDO1* TaqMeth V tended to correlate inversely with the time to development (r^2^ = 0.06, *P* = 0.10), but the correlation was marginally significant. On the other hand, *HOPX* TaqMeth V correlated inversely but significantly with the time to development (r^2^ = 0.11, *P* = 0.03). Similarly, *Reprimo* TaqMeth V correlated negatively and significantly with the time to development (r^2^ = 0.14, *P* = 0.01). *E-cadherin* TaqMeth V did not correlate significantly with the time to development (r^2^ = 0.015, *P* = 0.43), but the graph showed evidence of a mild negative correlation.

Because the term to the development of RGC correlated inversely with TaqMeth V in biopsy specimens, whether a model could be designed to predict the short-term risk using TaqMeth Vs of the 4 genes in biopsy specimens was evaluated. First, the optimal cut-off TaqMeth Vs for dividing the time to development into a short-term group and a long-term group were calculated using Student’s *t-*test and ROC curves (Figure 5B). The optimal cut-off TaqMeth Vs for dividing the time to development into 2 groups were: 1.2 for *CDO1* (*P* = 0.02, sensitivity 50%, specificity 79%), with a mean time to development of 65 ± 60 months in the short-term group (*n* = 31) and 127 ± 103 months in the long-term group (*n* = 12); 0.60 for *HOPX* (*P* = 0.0009, sensitivity 87%, specificity 42%), with a mean time to development of 62 ± 51 months in the short-term group (*n* = 33) and 152 ± 113 months in the long-term group (*n* = 10); 0.15 for *Reprimo* (*P* = 0.001, sensitivity 71%, specificity 75%), with a mean time to development of 51 ± 39 months in the short-term group (*n* = 15) and 126 ± 98 months in the long-term group (*n* = 18); and 0.07 for *E-cadherin* (*P* = 0.07, sensitivity 29%, specificity 83%), with a mean time to development of 41 ± 27 months in the short-term group (*n* = 9) and 94 ± 84 months in the long-term group (*n* = 34). Using the cutoff value of each gene, significant differences were obtained in the period until onset, and then we performed multivariate analysis. The results showed that *HOPX* was an independent factor (*P* = 0.02) (*CDO1*, *P* = 0.94; *Reprimo*, *P* = 0.10; *E-Cadherin*, *P* = 0.22).

These results showed that the term to development of RGC could be divided into 2 groups according to the TaqMeth V of each gene. A model for predicting short-term risk was developed by including the cut-off TaqMeth Vs. TaqMeth Vs less than the cut-off value were scored as 0 points, and TaqMeth Vs equivalent to or higher than the cut-off value were scored as 1 point. The time to development was evaluated using combinations of the TaqMeth Vs of the 3 genes that showed strong correlations with the term to development (*CDO1, HOPX,* and *Reprimo*), as well as with all 4 genes, including *E-cadherin* (*CDO1*, *HOPX*, *Reprimo*, and *E-cadherin*) (Table 2). When combinations of all 4 genes were included and the biopsy specimens were evaluated from a minimum of 0 points to a maximum of 4 points, the mean term to the development of RGC was 140 ± 98 months in the patients with a score of 0 or 1 (*n* = 16) and 49 ± 35 months in the patients with a score of 2 to 4 (*n* = 27), and the most significant difference was seen between the 2 groups (*P* < 0.0001) (Figure 5C).

**Table 2:** Evaluation in combination the scores of each gene

Combination	Score	*P*-value
*CDO1, HOPX*	0, 1, 2	0.003
	0, 1–2	0.01
	0–1, 2	0.0009
*CDO1, Reprimo*	0, 1, 2	0.002
	0, 1–2	0.03
	0–1, 2	0.0004
*HOPX, Reprimo*	0, 1, 2	0.0004
	0, 1–2	0.0005
	0–1, 2	0.0008
*CDO1, HOPX, Reprimo*	0, 1, 2, 3	0.003
	0, 1–3	0.02
	0–1, 2–3	0.0004
	0–2, 3	0.0008
*CDO1, HOPX, Reprimo, E-Cadherin*	0, 1, 2, 3, 4	0.003
	0, 1–4	0.003
	0–1, 2–4	<0.0001
	0–2, 3–4	0.001
	0–3, 4	0.43

Finally this scoring system was applied to the RN in our 58 RGC series. Among the 35 RGC with initial diagnosis of malignant disease, 2 patients (5.7%) had a score of 0, 6 (17.1%) had a score of 1, 14 (40%) had a score of 2, 10 (28.6%) had a score of 3, and 3 (8.6%) had a score of 4. As a result, patients with a score of 2 or higher were found in 27 (77.1%) from the 35 patients. On the other hand, the methylation score in the remnant gastric non-cancerous mucosa was 2 or higher in 13 (56.5%) of the 23 RGC patients with initial diagnosis of benign disease, and the rates between RGC patients with initially malignant disease and those with initially benign disease were significant difference (*P* < 0.0001).

## DISCUSSION

In this current study, we investigated 4 cancer-prone methylation genes in 5 types of non-cancerous mucosa tissues as well as RGC tumor tissues in comparison with primary gastric cancer [17, 19, 20]. The cut-off TaqMeth values of *CDO1*, *HOPX*, and *Reprimo* obtained from comparison between RGC tumor tissues and the RN were very similar with those primary ones [19, 20], indicating that pin-point dense methylation event may result in cancer formation of both RGC and primary gastric cancer.

As previous reports with regard to RGC [7, 31], there is huge difference of the interval term from the initial operation to the RGC onset between patients with initial benign disease and those with initial malignant disease (486 months vs 192 months in this study). This data suggested that long-term follow-up periods after surgery are required for RGC surveillance, which must be an obstacle for good compliance, and risk assessment of RGC onset has therefore become an urgent issue in gastric cancer clinics. In this study, a model for predicting the RGC risk was for the first time developed. DNA methylation assay using the 4 cancer-prone methylation genes in the RN-Biopsy by endoscopy is likely to be helpful to predict the RGC onset in the near future (Figure 5C).

Our current scoring system was also applied to the RN in addition to RN-Biopsy by endoscopy. In RN-IM, methylation with a score of 2 or higher had occurred in 77.1%, while it was seen in 56.5% in RN-IB. The methylation scores between RN-IM and RN-IB showed statistical difference between RN-IM and RN-IB (*P* < 0.0001). Moreover, *HOPX* hypermethylation is recognized uniquely in RN with intestinal type histology (Table 1). These findings suggested that field cancerization of RGC is deemed prone to intestinal type histology, and *HOPX* plays an important role in epigenetic field cancerization in RN-IM. *HOPX* hypermethylation also tended to be associated with HP infection in both RGC and RN (*P* = 0.06 and *P* = 0.1), suggesting that *HOPX* methylation with its gene silencing may play a critical role in HP-induced intestinal metaplasia-adenocarcinoma sequence.

Allowing for IN-IM/IB, on the other hand, *CDO1*/*HOPX* hypermethylation is recognized in IN-IM, in comparison with IN-IB or RN-IB/IM. This finding also indicated that field cancerization hypothesis for gastric carcinogenesis [32, 33] may be characteristic to IN-IM rather than RN-IM. Hence DNA methylation assay in the non-cancerous mucosa tissues (IN-IM in this study) would be helpful to predict onset even of primary gastric cancer in addition to RGC. A retrospective study to confirm this hypothesis in primary gastric cancer are being conducted at present.

In our analysis, patients with initial benign disease occurred RGC significantly much later than patients with initial malignant disease. This result was consistent with the existing report [7, 31]. DNA methylation is well known to occur prior to carcinogenesis as shown in our current study (Figure 1); risk accumulation surrounding non-cancerous mucosa tissues is thought to cause cancer, which is called field cancerization. In the case of patients with initial malignant disease, the initial non-cancerous gastric mucosa tissues that had surrounded the initial cancer are thought to harbor methylation events, especially for *CDO1*, that are risky for remnant gastric cancer (Figure 3, Supplementary Figure 4). On the other hand, in the case of patients with benign disease, the initial gastric mucosa tissues that had surrounded the initial benign disease are thought to harbor less methylation events than the initial malignant disease (Figure 3, Supplementary Figure 4). This may be the reason explaining huge difference of duration of remnant cancer from initial surgery between the initial malignant and the initial benign patients.

In RGC tumors, on the other hand, *CDO1* hypermethylation is significantly associated with advanced disease stage, and *E-cadherin* hypermethylation was associated with advanced lymph node metastasis (Table 1), which are consistent with previous studies of primary gastric cancer [34, 35]. *CDO1* and *HOPX* hypermethylation in RGC was also a prognostic factor as well as other known clinicopathological factors in RGC [36], which is again consistent with the previous results of *HOPX* [19]. These findings strongly supports the hypothesis that *CDO1*, *HOPX,* and *E-cadherin* are potent tumor suppressor genes in gastric cancer. The 3 molecules are involved in Wnt pathway inhibition in the numerous studies, which are consistent with the brand new hypothesis that Wnt pathway is a critical determinant for gastric carcinogenesis [37].

*E-cadherin* methylation rate was much lower than expected, while it was correlated with lymph node metastasis in RGC tumors, which recapitulates the previous reports [38]. Moreover, *E-cadherin* hypermethylation is observed in IN-IB in contrast to IN-IM or RN. This finding is also consistent with the previous report describing that HP infection affects *E-cadherin* mRNA expression through modification of its promoter DNA methylation [39, 40]. In terms of the *E-cadherin* methylation data, we have to note that its methylation values (TaqMeth V∼10) is much lower than those (TaqMeth V∼100) of other 3 genes.

For methylation analysis of *E-cadherin*, we initially explored cell lines which expressed *E-cadherin* (Supplementary Figure 5A). Cancer cell lines expressing *E-cadherin* mRNA did not have methylation (MCF7, MKN74), while those expressing no *E-cadherin* mRNA had hypermethylation (SH10) in direct sequencing for promoter DNA (Supplementary Figure 5B, 5C). For MCF7 (E-cadherin mRNA expression) and SH10 (no *E-cadherin* mRNA expression), cloned sequence confirmed the fine status of promoter DNA methylation level (1.8% methylation in MCF7, and 90.5% methylation in SH10) (Supplementary Figure 5D). These findings suggested that *E-cadherin* mRNA is consistent with *E-cadherin* promoter DNA methylation. Using SH10 DNA as a positive control of DNA methylation, real-time quantitative TaqMan methylation-specific PCR (Q-MSP) was originally developed, and PCR affinity is relatively low (Supplementary Figure 5E) as compared to beta-actin or other 3 genes. That is why low level of *E-cadherin* methylation TaqMeth V is observed in RGC tumor tissues (Figure 1A, the right panel).

Limitation. In our hospital, 2268 patients underwent gastrectomy for gastric cancer from 2001 through 2014. Of these patients, 58 (2.6%) underwent surgery for RGC. The incidence of RGC is thus relatively low, and a long interval is required for its development. The specimens used in the present study therefore served as a training set. We are planning to evaluate the appropriateness of our model with the use of a validation set by performing a randomized, prospective study.

In conclusion, the models advocated by us can make the surveillance after gastrectomy more efficient. And this could lead to a reduction in burden on patients and medical economics. Very promising results were obtained for patients with remnant stomach.

## MATERIALS AND METHODS

### Patients and tissue samples

The study consisted of 58 RGC patients who underwent total gastrectomy, lymph node dissection, and Roux-en-Y anastomosis in Kitasato University Hospital from 2001 through 2014. A total of 58 pairs of cancer tissues and corresponding non-cancerous mucosa tissues were retrospectively identified. In order to compare the non-cancerous mucosa in the initial gastrectomy (for the initial malignant disease) with that in RGC, 17 patients were available. On the other hand, non-cancerous mucosa of the 12 patients who had undergone distal gastrectomy for gastric or duodenal ulcer (benign disease) between 1986 and 2000 was investigated.

To investigate time-dependent changes, the biopsy specimens obtained by upper gastrointestinal endoscopy were also used. The biopsy specimens were 43 specimens from the 12 patients whose initial diagnoses were only malignant and they underwent initial gastrectomy in our hospital.

Two patients with RGC had received preoperative chemotherapy, and 17 patients had received postoperative chemotherapy.

All samples that were formalin-fixed and paraffin-embedded were used. Informed consent to use the specimens was obtained from all patients. This study was conducted in accordance with the Declaration of Helsinki, and approved by the Kitasato University Medical Ethics Organization.

### Clinicopathological factors

Clinicopathological factors included age, sex, initial diagnosis, HP infection, term from initial operation to diagnosis of the RGC, reconstruction of the initial operation, recurrence site, Lauren’s histological type, depth of tumor invasion (T), lymph-node metastasis (N), distant metastasis (M), staging classification, lymphatic invasion (ly) and vascular invasion (v) according to the 7th edition of the American Joint Committee on Cancer/International Union Against Cancer (7th UICC) staging system, and the infiltrative growth pattern (INF) was judged according to the 14th Japanese Classification of Gastric Carcinoma.

### Biopsy specimens

Biopsy was performed an average of 83.6 (± 78.5) months before the development of RGC. Biopsy was performed because endoscopy revealed the presence of mucosal redness or erosion associated with gastritis of the remnant stomach. The histopathological diagnosis of the biopsy tissue was acute or chronic inflammation (or both) or regenerating mucosa.

### DNA purified from tissues and bisulfite treatment of DNA

Tissue sections from tumor, corresponding normal mucosa, and biopsy specimens were sharply dissected on hematoxylin and eosin-stained slides. Genomic DNA was subsequently extracted using of a QIAamp DNA FFPE Kit (Qiagen Sciences, Hilden, Germany). Bisulfite treatment was carried out using an EZ DNA Methylation-GoldTM Kit (Zymo Research, Orange, CA, USA).

### Quantitative methylation-specific PCR

Q-MSP was carried out using iQ Supermix (Bio-Rad, Hercules, California, USA) in triplicate on the iCycler iQTM Real-Time PCR Detection system (Bio-Rad). Q-MSP was done at 95° C for 3 min, followed by 40 cycles at 95° C for 20 sec, annealing temperature for 30 sec, and 72° C for 30 sec in a 25-μL reaction volume containing 1 μL of bisulfite-treated genomic DNA, 300 nmol/L of each primer, 200 nmol/L of fluorescent probe, and 12.5 μL of iQTM Supermix. Methylation-positive and -negative controls, PCR conditions, and sequences of primer and probe are provided in Supplementary Table 4 [41] and Supplementary Figure 5. The methylation value (TaqMeth V) was defined as the quantity of fluorescence intensity derived from promoter amplification of the positive control gene divided by the fluorescence intensity from β-actin and then multiplied by 100.

### Statistical analysis

The TaqMeth V of each target gene and clinicopathological factors was compared in Student’s *t*-test, the chi-squared test, Tukey’s honestly significant difference test, and variance, if appropriate. Five-year OS and RFS were calculated by the Kaplan-Meier method, and statistical differences were tested by the log-rank test. OS and RFS were calculated from the date of surgery of the RGC to the date of events or the last follow-up. Variables suggested to be prognostic factors on univariate analysis (*P* < 0.05) were subjected to multivariate analysis using a Cox proportional-hazards regression model. *P* < 0.05 was considered significant. All statistical analyses were performed with SAS software package JMP, version 11 (SAS Institute INC., Cary, NC, USA).

## SUPPLEMENTARY MATERIALS FIGURES AND TABLES



## Abbreviations

RGC: remnant gastric cancer
HP: Helicobacter pylori
CDO1: cysteine dioxygenase 1
HOPX: homeodomain-only protein X
OS: overall survival
RFS: relapse-free survival
RN-IM: remnant gastric non-cancerous mucosa from patients with an initial diagnosis of malignant disease
RN-IB: remnant gastric non-cancerous mucosa from patients with an initial diagnosis of benign disease
IN-IM: initially obtained specimens of non-cancerous mucosa from patients with an initial diagnosis of malignant disease
IN-IB: initially obtained specimens of non-cancerous mucosa from patients with an initial diagnosis of benign disease
RN: remnant gastric non-cancerous mucosa
ROC: receiver operating characteristic
Q-MSP: quantitative TaqMan methylation-specific PCR
T: tumor invasion
N: lymph-node metastasis
M: distant metastasis
ly: lymphatic invasion
v: vascular invasion
UICC: the American Joint Committee on Cancer/International Union Against Cancer
INF: infiltrative growth pattern
TaqMeth V: methylation value
