# Supplementary material for: Prediction of onset of remnant gastric cancer by promoter DNA methylation of CDO1/HOPX/Reprimo/E-cadherin
Source: Oncotarget. 2019 Mar 29;10(25):2423–34. doi: 10.18632/oncotarget.26814 (PMC6497431; doi:10.18632/oncotarget.26814)
Supplement: Supplementary file 1 [file oncotarget-10-2423-s001.pdf]

Prediction of onset of remnant gastric cancer by promoter DNA methylation of CDO1/HOPX/Reprimo/E-cadherin

SUPPLEMENTARY MATERIALS

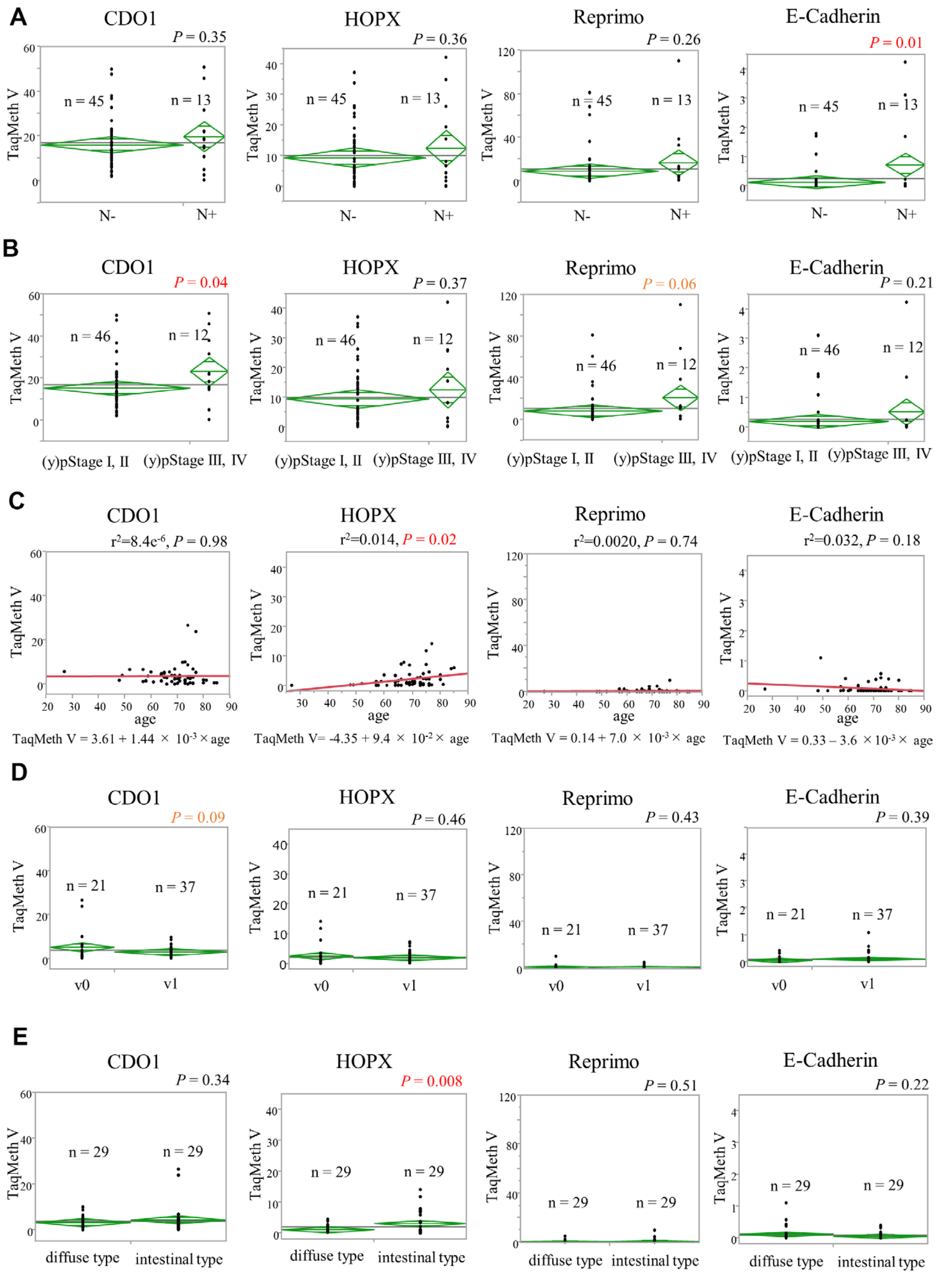

Supplementary Figure 1: Associations of clinicopathological factors with TaqMeth V in the remnant gastric cancer tissue and remnant stomach non-cancerous mucosa. (**A**) The relationships between *CDO1*, *HOPX*, *Reprimo*, and *E-cadherin*, and lymph node metastasis in remnant gastric cancer tissue. (**B**) The relationships between *CDO1*, *HOPX*, *Reprimo,* and *E-cadherin* and (y)pStage in remnant gastric cancer tissue. (**C**) The relationships between *CDO1*, *HOPX*, *Reprimo*, and *E-cadherin* and age in non-cancerous mucosa of remnant gastric cancer. (**D**) The relationships between *CDO1*, *HOPX*, *Reprimo*, and *E-cadherin* and venous invasion in non-cancerous mucosa of remnant gastric cancer. (**E**) The relationships between *CDO1*, *HOPX*, *Reprimo*, and *E-cadherin* and histological type in non-cancerous mucosa of remnant gastric cancer.

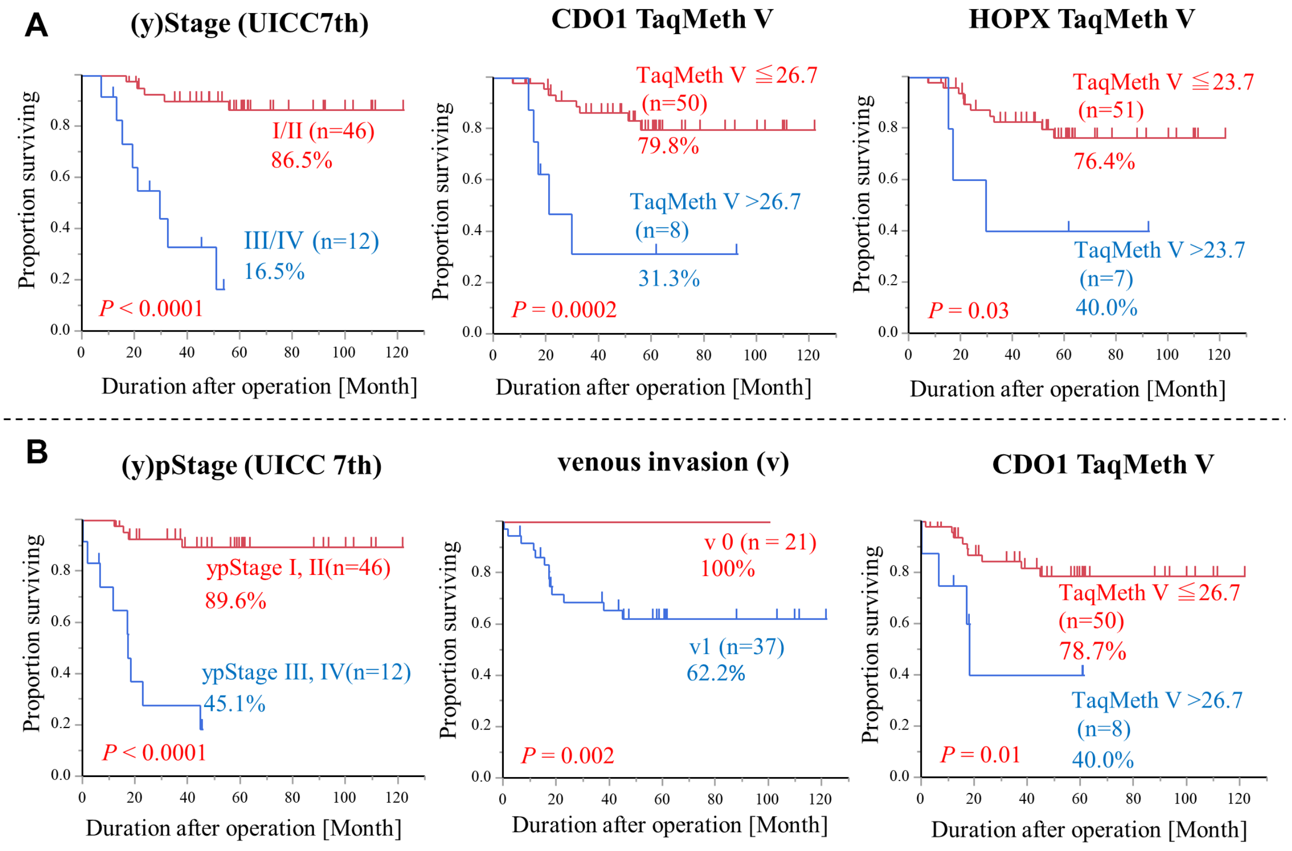

Supplementary Figure 2: Prognostic analysis of remnant gastric cancer. (**A**) OS of remnant gastric cancer patients according to the (y)pStage, *CDO1* TaqMeth V, and *HOPX* TaqMeth V using Kaplan-Meier survival curves. (**B**) RFS of remnant gastric cancer patients according to the (y)pStage, venous invasion, and *CDO1* TaqMeth V using Kaplan–Meier survival curves.

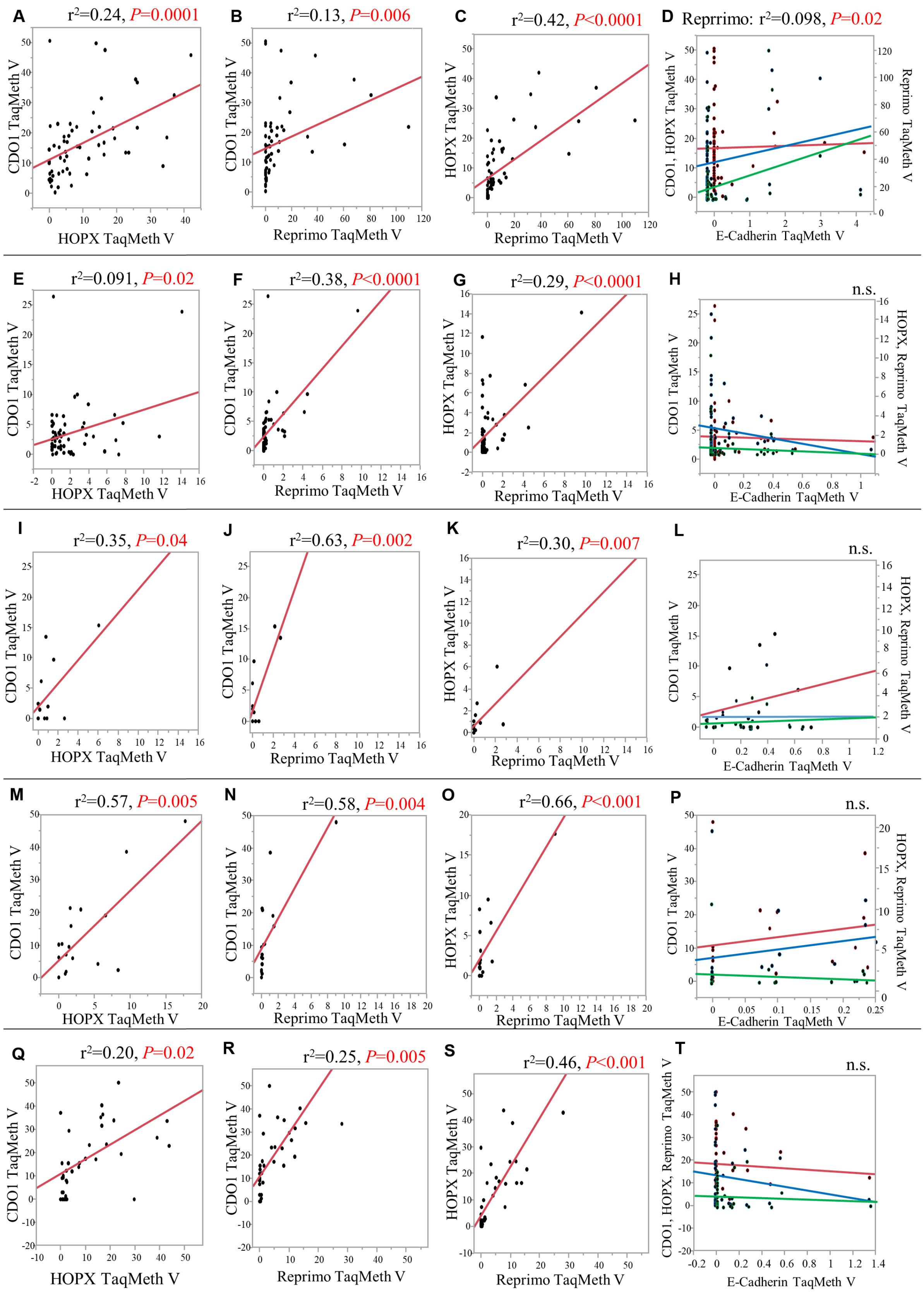

Supplementary Figure 3: Inter-relationships among each of the TaqMethVs of 4 genes. (**A**–**D**) Correlations in the remnant gastric cancer tissue. (**E**–**H**) Correlations in the non-cancerous mucosa of remnant stomach. (**I**–**L**) Correlations in the non-cancerous mucosa of initial surgery that was diagnosed with benign disease at the initial diagnosis. m-p: Correlations in the cancer tissue of initial surgery that was diagnosed with malignant disease at the initial diagnosis. (**Q**–**T**) Correlations in the biopsy specimen. (A, E, I, M and Q) Correlations of *CDO1* with *HOPX*. (B, F, J, N and R) Correlations of *CDO1* with *Reprimo*. (C, G, K, O and S) Correlations of *HOPX* with *Reprimo*. (D, H, L, P and T) Correlations of *E-cadherin* with *CDO1*, *HOPX* and *Reprimo*. The relationship between *CDO1* and *E-Cadherin* is shown by the red line. The relationship between *HOPX* and *E-Cadherin* is shown by the blue line. The relationship between *Reprimo* and *E-cadherin* is shown by the green line.

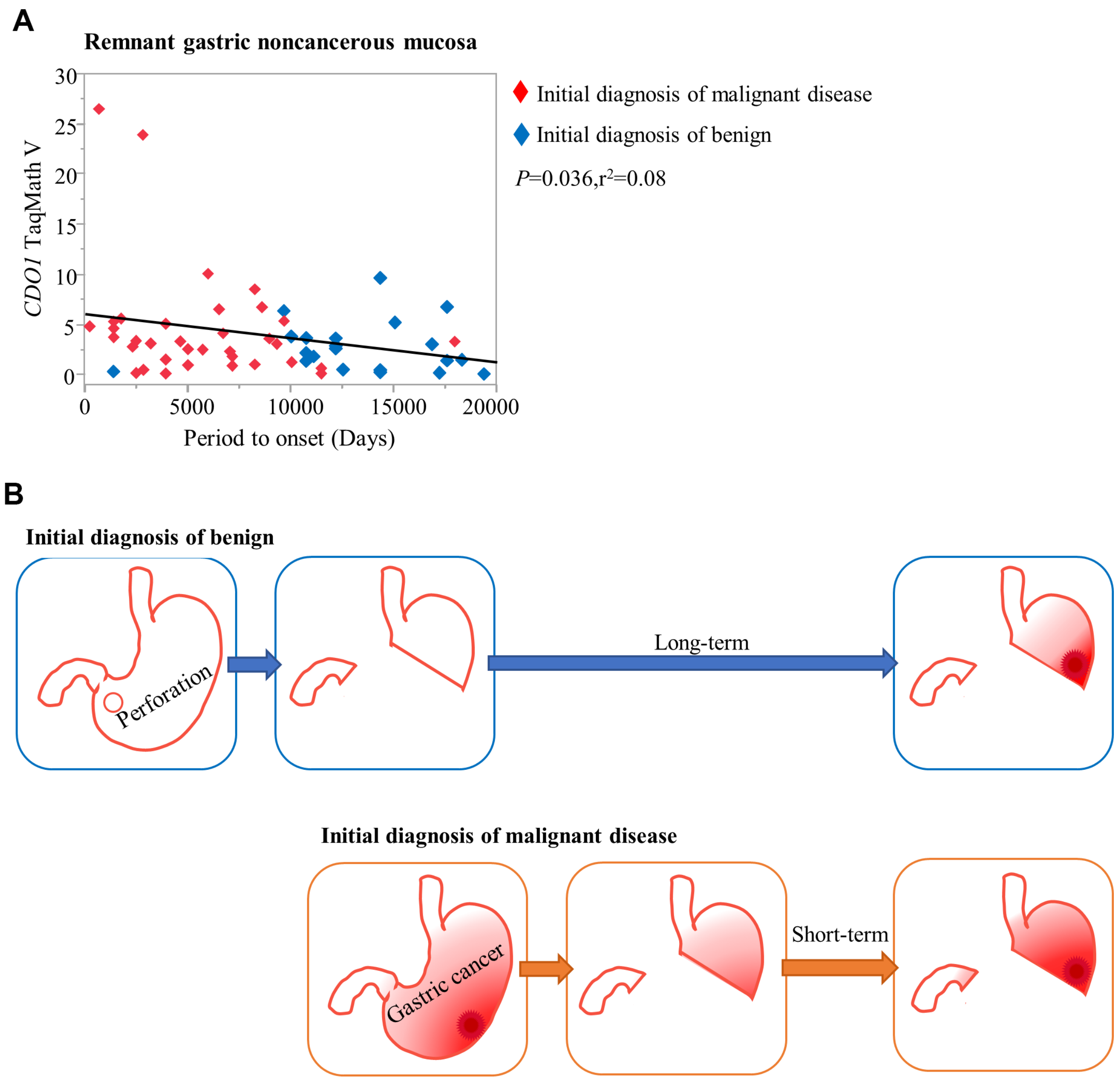

Supplementary Figure 4: Schematic of a consideration on the difference up to the onset of RGC. (**A**) The relationship between *CDO1* TaqMeth V in the remnant gastric non-cancerous mucosa (RN) and the period until the last surgery is shown. The shorter the period from the initial surgery, the higher the methylation abnormality of the RN tended to be. Red points are initial malignant diseases. Blue points are initial benign diseases. (**B**) Schematic of accumulation of methylation occurring in non-cancerous mucosa of each initial disease. In the initial benign diseases, the accumulation of methylation abnormality is not conspicuous in non-cancerous mucous membranes. Methylation abnormality accumulates in the RN after a long term, resulting in carcinogenesis. On the other hand, in initial malignant diseases, accumulation of methylation abnormality has already occurred in part of the RN. Therefore, there is a possibility of developing RGC in a short time.

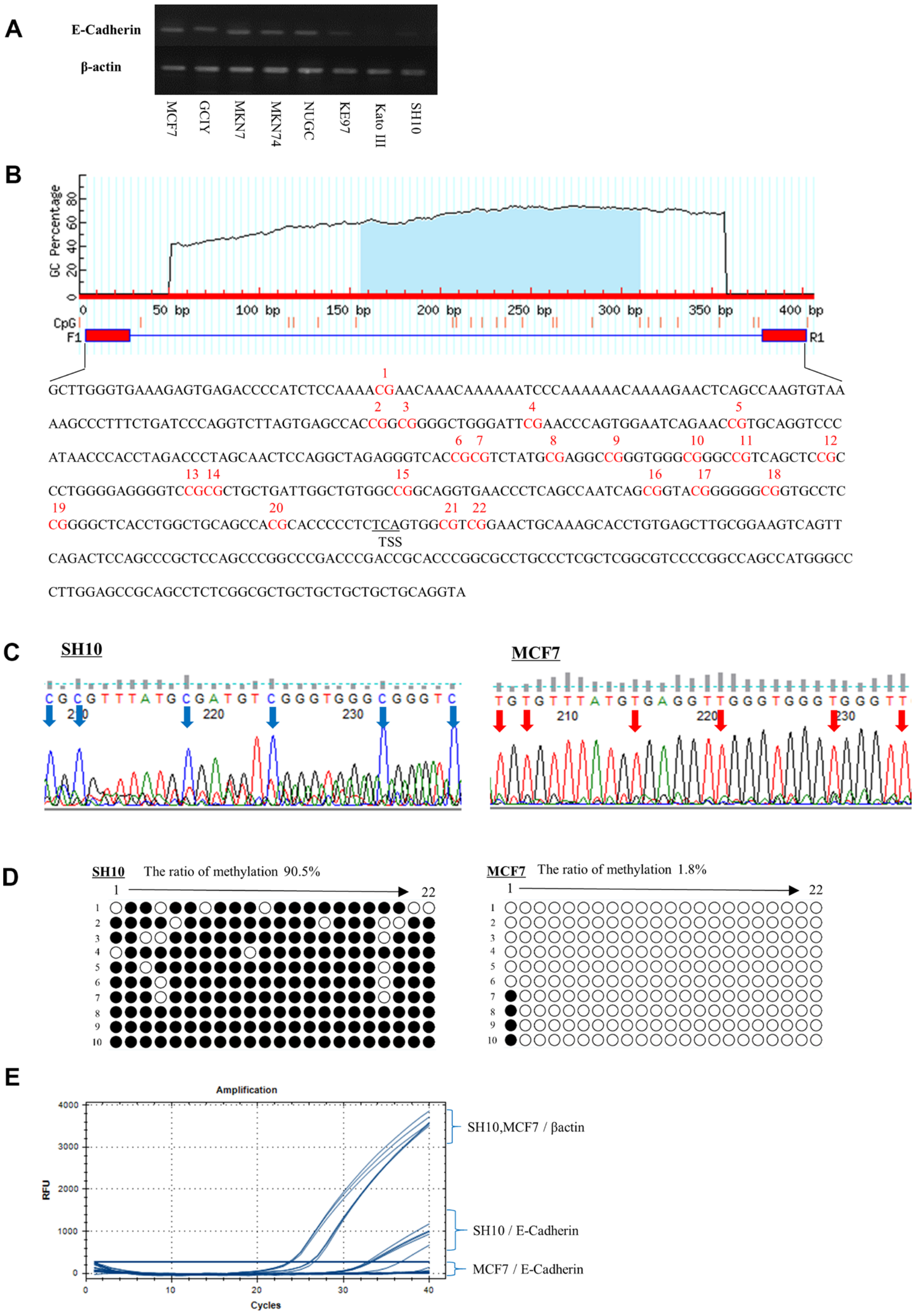

Supplementary Figure 5: Basis to determine the control of *E-cadherin* for Q-MSP. (**A**) Results of the expression levels of *E-cadherin* in gastric cancer cell lines and MCF7 examined by reverse transcriptase-PCR (RT-PCR). Expressions in SH10 and Kato III were suppressed, and KE97 was weak; other cell lines were expressed. (**B**) Primer creation area of bisulfite-sequencing. An array of the upstream and downstream of the transcription start site (TSS), including primers and probe known Q-MSP of *E-cadherin* [41]. Primers for bisulfite sequencing were designed to have a CpG-rich region including 22 CGs. The CGs are numbered in order. (**C**) Representative results of *E-cadherin* bisulfite-sequencing in SH10 and MCF7. Methylated cytosine is not converted (blue arrow), only the non-methylated cytosine is represented as thymine (red arrow). When cell lines are bisulfite sequenced, there is a high degree of methylation in SH10 and unmethylated in MCF7. (**D**) Cloned PCR products from SH10 and MCF7. White and black circles denote unmethylated and methylated CpG sites, respectively. The proportion of methylation is 90.5%in SH10 and 1.8% in MCF7. For the cloned sequence analysis, the PCR products were gel-extracted using the QIAquick PCR Purification Kit (QIAGEN) and inserted into the pCR4-TOPO vector using a TOPO TA cloning kit for sequencing (Invitrogen, Carlsbad, CA, USA); 10 clones were selected for each sample and then sequenced. Sequence analysis was carried out at eurofins Genomics K. K. (**E**) Results of the Q-MSP of SH10 and MCF7. The mean TaqMeth V of SH10 is 1.8, and that of MCF7 is 0. In addition, the mean threshold cycle of SH10 is 31.5, and that of MCF7 is 0.

Supplementary Table 1: Clinicopathological characteristics of remnant gastric cancer (*n* = 58)

Factor	Initial diagnosis		*P*-value
	Benign (*n* = 23)	Malignancy (*n* = 35)	
Sex			0.23
Male	22	29	
Female	1	6	
Age(year)			0.38
Mean ± S.D.	70.1 ± 7.9	67.8 ± 10.5	
Median(range)	71 (49–85)	70 (27–84)	
Interval between initial operation to diagnosis(month)			<0.0001
Mean ± S.D.	485.7 ± 86.7	191.5 ± 127.5	
Median(range)	450 (48–648)	168 (9–600)	
Initial gastrectomy method			0.15
Distal gastrectomy	23	32	
Proximal gastrectomy	0	3	
Reconstruction of initial operation			<0.0001
BI	8	30	
BII	14	2	
Roux-en-Y	1	0	
Jejunal interposition	0	3	
Location			0.03
Anastomotic site	16	14	
Non-anastomotic site	7	21	
ypT factor(7th UICC)			0.07
T1a(M)	1	8	
T1b(SM)	5	15	
T2(MP)	5	4	
T3(SS)	2	1	
T4a(SE)	8	6	
T4b(SI)	2	1	
ypN factor(7th UICC)			0.04
N0	16	29	
N1(1~2)	3	1	
N2(3~6)	1	5	
N3a(7~15)	3	0	
N3b(16~)	0	0	
ypM factor(7th UICC)			0.51
M0	23	33	
M1	0	2	
ypStage(7th UICC)			0.02
IA	5	20	
IB	5	7	
IIA	1	1	
IIB	5	2	
IIIA	1	0	
IIIB	3	3	
IIIC	3	0	

IV	0	2	
Lymphatic invasion (ly) (7th UICC)			<0.0001
0	2	21	
1	21	14	
Venous invasion (v) (7th UICC)			0.003
0	3	18	
1	20	17	
Lauren's histology			0.59
Intestinal type	10	16	
Diffuse type	13	19	
Infiltrative pattern (14th JGC)			0.03
a	5	20	
b	10	8	
c	8	7	

Supplementary Table 2: Univariate and multivariate prognostic analysis of clinicopathological factors for OS in remnant gastric cancer

Clinicopathological parameters	Categories	account	Univariate analysis		Multivariate analysis		
			5year OS(%)	*P*-value	Hazard ratio	95% CI	*P*-value
Age	≤70	29	75.6	0.62			
	70<	29	69.8				
Gender	male	51	72.5	0.81			
	female	7	75				
Initial diagnosis	Benign	23	62	0.13			
	Malignancy	35	81.4				
Site of recurrence	anastomotic site	30	77.1	0.37			
	Other sites	28	67.2				
Reconstruction method of initial surgery	Billroth I	38	81.8	0.13			
	Billroth II	16	52				
	Other method	4	- a				
Depth of tumor invasion	pT1	29	96.2	0.0007			
	pT2-pT4	29	50				
Lymph node metastasis	pN0	45	81	0.009			
	pN1-pN3	13	46				
Distant metastasis	cM0	56	75.9	<0.0001			
	cM1	2	0				
(y)pStage	pStage I–II	46	86.5	<0.0001	6.96	1.52–37.1	0.01
	pStage III–IV	12	16.5				
Lymphatic permeation (ly)	ly0	23	95.2	0.009	3.04	0.38–65.1	0.32
	ly1	35	58.5				
Vascular permeation (v)	v0	21	94.4	0.03	2.56	0.36–52.5	0.38
	v1	37	62.5				
Lauren's histological type	intestinal type	29	77.5	0.41			
	diffuse type	29	68.5				
Infiltrative growth pattern (INF)	INF a and b	43	83.6	0.002	1.16	0.25-5.30	0.49
	INF c	15	32.2				
CDO1 TaqMeth Value (cancer tissue)	≤26.7	50	79.8	0.0002	3.78	0.59–19.3	0.15
	26.7<	8	31.3				
HOPX	≤23.7	51	76.4	0.03	0.84	0.13–5.46	0.84
	23.7<	7	40				

a: It can not be calculated.

Supplementary Table 3: Univariate and multivariate prognostic analysis of clinicopathological factors for RFS in remnant gastric cancer

Clinicopathological parameters	Categories	Account	Univariate analysis		Multivariate analysis		
			5year OS(%)	*P*-value	Hazard ratio	95% CI	*P*-value
Age	≤70	29	77.6	0.69			
	70<	29	69.8				
Gender	male	51	73.5	0.80			
	female	7	80				
Initial diagnosis	Benign	23	84.7	0.03	2.03	0.58–8.77	0.28
	Malignancy	35	60.1				
Site of recurrence	anastomotic site	30	81.9	0.19			
	Other sites	28	65				
Reconstruction method of initial surgery	Billroth I	38	82.3	0.13			
	Billroth II	16	50				
	Other method	4	- a				
Depth of tumor invasion	pT1	29	100	<0.0001			
	pT2-pT4	29	50.8				
Lymph node metastasis	pN0	45	84.5	0.002			
	pN1-pN3	13	42				
Distant metastasis	cM0	56	76.9	<0.0001			
	cM1	2	0				
(y)pStage	pStage I–II	46	89.6	<0.0001	9.20	2.13–47.71	0.003
	pStage III–IV	12	45.1				
Lymphatic permeation (ly)	ly0	23	100	0.002	1.27E+09	0.46–3.73E+39	0.15
	ly1	35	58.3				
Vascular permeation (v)	v0	21	100	0.007	1.25E+09	1.23—	0.03
	v1	37	62.2				
Lauren's histological type	intestinal type	29	83.3	0.13			
	diffuse type	29	64.8				
Infiltrative growth pattern (INF)	INF a and b	43	87	0.0003	0.86	0.20–3.61	0.84
	INF c	15	30.5				
CDO1 TaqMeth Value (cancer tissue)	≤26.7	50	78.7	0.01	1.61	0.40–5.61	0.48
	26.7<	8	40				

a: It can not be calculated.

Supplementary Table 4: PCR condition and primer and fluorescent probe sequences for quantitative taqman methylation-specific PCR (Q-MSP)

Gene	Forward primer	Fluorescent probe or internal primar	Reverse primer	Tm[a]	Positive control	Negative control
HOPX	5′-TTTGGAGAGGGTT TTAAAGCG-3′	FAM-5′-CGGAGATAGAAGGTCG TTTATCGGGGAGGTCG-3′TAMRA	5′-AACAAACTTAA CAAATCGCGAA-3′	60	KatoIII	TE15
CDO1	5′-CCACAACGACGAAA ATAAAACG-3′	FAM-5′-TTAACGGCGCGTTTTAG TCGTTCG-3′TAMRA	5′-TCGGCGTTTT AGGGATCGCG-3′	60	DLD1	HepG2
Reprimo	5′-GCGGTCGGA AGGGGTC-3′	FAM-5′-TTAAAACTTAACGAAAC TAAACCAACCCGACCGT-3′TAMRA	5′-ACTCGTTCCC GACGCTCG-3′	65	KatoIII	GCIY
E-Cadherin	5′-GAATTAGAATCGTGTAG GTTTTATAATTTATTTAGA-3′	FAM-5′-ACCTCGCATAAACGC GATA-3′MGBNFQ	5′-CCGACCACAA CCAATCAACA-3′	54	SH10	MCF7
β-actin	5′-TGGTGATGGAGGA GGTTTAGTAAGT-3′	FAM-5′-ACCACCACCCAACACA CAATAACAAACACA-3′TAMRA	5′-AACCAATAAAACCT ACTCCTCCCTTAA-3′			

a: Annealing temperature.
